# Immune reconstitution from peripheral blood mononuclear cells inhibits lung carcinoma growth in NOD/SCID mice

**DOI:** 10.3892/ol.2014.2379

**Published:** 2014-07-24

**Authors:** XIANG LIU, HUILING LI, JUN LIU, YUBAO GUAN, LIYAN HUANG, HAILING TANG, JIANXING HE

**Affiliations:** 1 Department of Cardiothoracic Surgery, The Second Hospital Affiliated to University of South China, Hengyang, Hunan 421001, P.R. China; 2 Department of Cardiothoracic Surgery, Guangzhou Institute of Respiratory Disease, State Key Laboratory of Respiratory Disease, The First Affiliated Hospital of Guangzhou Medical College, Guangzhou, Guangdong 510120, P.R. China; 3 Department of Respiratory Medicine, Hainan Branch of Chinese PLA General Hospital, Sanya, Hainan 572000, P.R. China; 4 Department of Radiology, Guangzhou Institute of Respiratory Disease, State Key Laboratory of Respiratory Disease, The First Affiliated Hospital of Guangzhou Medical College, Guangzhou, Guangdong 510120, P.R. China

**Keywords:** NOD/SCID, T lymphocytes, peripheral blood mononuclear cells, lung carcinoma, Am1010

## Abstract

Drug resistance and immune deficiency are important factors for the poor prognosis of lung carcinoma. The present study explored the possible protective effect of immune reconstitution from peripheral blood mononuclear cells (PBMCs) on multi-drug-resistant human lung carcinoma Am1010 cells in non-obese diabetic/severe combined immunodeficient (NOD/SCID) mice. The inoculated tumor fragments grew rapidly in the NOD/SCID mice. The growth was significantly inhibited by intraperitoneal injection of PBMCs. In the mice injected with PBMCs, numerous CD3^+^ and CD8^+^ cells, but less CD4^+^ cells, were found in spleen and tumor tissues. These data suggest that PBMC transplantation inhibits lung carcinoma progression via the reconstitution of the immune system, particularly of cytotoxic T lymphocytes.

## Introduction

Lung cancer is the leading cause of cancer-related mortality worldwide (1). Numerous achievements have been made in chemotherapy, radiotherapy and surgical techniques. However, the survival rate has not been improved for many decades. This poor prognosis is mostly due to the development of drug resistance by cancer cells during treatment and the likelihood of subsequent metastasis (2). The drug-surviving cells (DSCs) are responsible for tumor regeneration after chemotherapy, and may be one of the mechanisms involved in drug resistance (3). Immunoresistance induced by chemotherapy agents may be another mechanism (4). Am1010, a new DSC line, was established in our previous study from an arm muscle metastasic tumor of a patient diagnosed with lung adenocarcinoma (5). The Am1010 cell line demonstrated *in vitro* multi-drug-resistance against cisplatin, taxol and geitinib, as well as *in vivo* invasion ability (5).

Chemotherapy of lung cancer is targeted not only at the tumor cells but also at the immune system. The immune system is a key factor in preventing the development of tumors and in limiting tumor growth (6). Tumor infiltration of lymphocytes is frequently found in tumors, suggesting that tumors trigger an immune response in the host. A number of studies have reported a survival benefit associated with the presence of tumor-infiltrating lymphocytes (TILs) (7–9); although, in some circumstances, it was a paradoxical result (10,11).

Non-obese diabetic/severe combined immunodeficient (NOD/SCID) mice have multiple defects in innate and adaptive immunologic functions (12). They are absent of T and B lymphocytes and natural killer (NK) cells. They also exhibit dysfunctional macrophages, dendritic cells and complement systems. Thus, NOD/SCID mice are an ideal tool for immunodeficient studies. These mice are also commonly used in studies involving various types of cancer xenografts (13,14). Previously, intravenous injection of granulocyte colony-stimulating factor-induced white blood cells from human peripheral blood into NOD/SCID mice was shown to produce a 6.5-month detection of human T lymphocytes (15). Peripheral blood mononuclear cell (PBMC) injection was reported to inhibit the growth of various types of tumors in NOD/SCID mice, including Burkitt’s lymphoma (16) and neuroblastoma cells (17). However, the protective role of PBMCs in lung cancer is not yet known to date.

In the present study, NOD/SCID mice were inoculated with lung carcinoma fragments (obtained by injecting Am1010 cells into NOD/SCID mice, allowing the tumors to grow to 150 mm^3^ and then cutting the tumors into equally sized fragments) and human PBMCs. An ideal heterotopic lung cancer model was thus established. Using the model, we observed the protective effect of PBMCs on tumor growth and the reconstitution of immune system.

## Materials and methods

### Reagents

Matrigel and FACS lysing solution were purchased from BD Biosciences (San Jose, CA, USA). Hydrogen peroxide (H_2_O_2_), 3,3′-diaminobenzidine and pentobarbital sodium were purchased from Sigma-Aldrich (St. Louis, MO, USA). RIPA lysis buffer and enhanced chemiluminescence (ECL) reagent were purchased from Pierce Biotechnology, Inc. (Rockford, IL, USA). Rabbit polyclonal antibody against PC5-CD3 was purchased from Beckman Coulter, Inc. (Brea, CA, USA). Rabbit monoclonal antibody against CD3 (ab109531), and rabbit polyclonal antibodies against CD4 (ab70951), CD8 (ab85792) and FoxP3 (ab10563) were purchased from Abcam (Cambridge, MA, USA). Rabbit polyclonal antibody against GAPDH was purchased from Cell Signaling Technology, Inc. (Danvers, MA, USA). Horseradish peroxidase (HRP)-coupled goat polyclonal anti-rabbit secondary IgG (sc-2004) and biotinylated goat polyclonal anti-rabbit IgG (sc-2040) secondary antibodies, as well as the avidin-biotin-HRP complex were purchased from Santa Cruz Biotechnology, Inc. (Dallas, TX, USA). RPMI-1640 medium and fetal bovine serum (FBS) were purchased from Life Technologies (Grand Island, NY, USA).

### Animals

Male NOD/SCID mice were provided by Experimental Animal Center, Sun Yat-Sen University (Guangzhou, China). The mice were kept in separate cages in a room with specific pathogen-free standards at a constant humidity and temperature, with food and water available *ad libitum*. The animal room was on a 12/12-h light/dark cycle. At the age of 4 weeks, blood samples were taken from the tail vein for determination of immunoglobulin (Ig) levels. Only the mice with IgM levels <1 μg/ml were used for further study. The experiments were performed at the 6th week. Tumor fragments were obtained by injecting Am1010 cells into six NOD/SCID mice, allowing the tumors to grow to 150 mm^3^ and then cutting the tumors into equally sized fragments. For the inoculation with tumor fragments and/or PBMCs, the mice were divided randomly into three groups. The Am1010+PBMC group was inoculated with a tumor fragment and PBMCs (n=10); the Am1010 group was inoculated with a tumor fragment (n=10); and the PBMC group was only injected with PBMCs (n=5). This study was approved by Guangzhou Medical College (Guangzhou, China).

### Establishment of xenografts

The green fluorescent protein (GFP)-Am1010 cell line was established in our previous study (5). The cells were stored in liquid nitrogen until use. A total of 2×10^7^ cells in a 200 μl volume of 50% Matrigel were injected subcutaneously into the dorsal surface of the right lower quadrant of the six NOD/SCID mice. Tumor growth was assessed by palpation every 3 days. The two bisecting diameters were measured with calipers, and the volume was calculated using the formula 0.4x*ab*^2^, where *a* represents the longer diameter and *b* the shorter perpendicular diameter (18). When the tumors grew up to a size of 150 mm^3^, they were removed and cut into 1×3×3 mm fragments. Twenty recipient mice were anesthetized with pentobarbital sodium (10 mg/kg). A 3-mm skin incision was cut at the sixth rib, left midaxillary line. A fragment of tumor was put into the incision, and the incision was sutured. The five other mice in the PBMC group underwent the same surgical procedure, but without insertion of a tumor fragment.

### Human PBMC preparation and transplantation

On the day of inoculation with tumor segments, human PBMCs were prepared according to a previous study (19). Fresh peripheral venous blood from healthy adult volunteers was collected at The First Affiliated Hospital of Guangzhou Medical College (Guangzhou, China) in heparinized tubes. For the isolation of PBMCs, leucosep tubes (Greiner Bio-One, Wemmel, Belgium) were used, and blood was diluted 1:1 with RPMI-1640 medium (vol/vol) prior to transferring into the leucosep tube. Following centrifugation (10 min, 1000 × g), the PBMC layer was pooled and transferred into a 15-ml falcon tube. The sample was washed with 10 ml phosphate-buffered saline (PBS) and centrifuged again for 10 min at 250 × g. The obtained cell pellet was resuspended in PBS. A total of 1×10^8^ PBMCs per mouse were intraperitoneally injected into NOD/SCID mice of the Am1010+PBMC and PBMC groups, for the reconstitution of immune system. The same volume of PBS was injected into the mice of the Am1010 group.

### Flow cytometry

To examine reconstitution of the immune system due to PBMC transplantation in the recipient NOD/SCID mice, CD3^+^ cells were analyzed by flow cytometry. In brief, peripheral blood (100 μl) was collected into EDTA-coated tubes from the tail vein at every week following the transplantation for four weeks. Red blood cells were first lysed with FACS lysing solution and then washed twice with PBS containing 2% FBS. The leukocytes were then incubated with PC5-labeled anti-CD3 antibody for 30 min at 4°C. The staining was assessed by flow cytometry. PBMCs obtained from normal human volunteers and normal mice were used as positive and negative controls, respectively. Results are expressed as the percentage of positive cells gated in the human lymphocyte population in the scatter plot.

### Whole-body fluorescence imaging

Whole-body fluorescence imaging was performed to examine the growth of the inoculated tumor fragment every seven days. The mice were anesthetized with 1% pentobarbital sodium (0.2 ml/20 g body weight), and were placed in a NightOWLII LB 983 molecular light imager (Berthold Technologies, Bad Wildbad, Germany). The images were photographed for 1 sec using a GFP filter (GFP Ex480/20 and Em520/10; Berthold Technologies). Data were processed with WinLight software (Winlight System, Pertuis, France) and the fluorescent area was recorded.

### Histological examination and immunohistochemistry

At four weeks frollowing tumor fragment and/or PBMC administration, all mice were anesthetized with 10% chloral hydrate and perfused intracardially with normal saline and 4% paraformaldehyde in 0.1 M phosphate buffer (pH 7.4). The spleens and tumors were fixed in 10% formalin solution, embedded in paraffin and cut serially into 4-μm sections. Some of the sections were stained with hematoxylin and eosin (H&E), while others were used for immunohistochemistry.

Immunohistochemistry was performed to detect CD3, CD4, CD8 and FoxP3 expression according to a previous study (20). Briefly, endogenous peroxidase was first quenched with 0.3% H_2_O_2_. Prior to the application of primary antibody, nonspecific binding was blocked with normal non-immune serum, and tissue sections were incubated with primary antibody (1:500 each) at 4°C overnight. Sections were then incubated with biotinylated secondary antibody (1:200) for 2 h at room temperature, followed by avidin-biotin-HRP complex (1:150) for 1 h. Immunohistochemical reactions were revealed by using 0.05% 3,3′-diaminobenzidine and 0.03% H_2_O_2_ as chromogen. Following each incubation, sections were thoroughly washed with PBS. Negative controls were performed by omitting the primary antibody, and showed no positive staining.

### Western blotting

Total tissue proteins were extracted with RIPA lysis buffer, quantified using the Bradford method and separated by SDS-PAGE (12%). A total of 40 μg of protein was used to test for CD3, CD4, CD8 and FoxP3. Proteins were transferred to polyvinylidene fluoride membranes (Millipore, Billerica, MA, USA), and membranes were incubated overnight at 4°C with antibody against CD3, CD4, CD8, FoxP3 (1:1000 each) or GAPDH (1:10,000). The membranes were incubated with HRP-coupled secondary IgG for 1 h. The bound proteins were then visualized using ECL and analyzed using BioImaging Systems (UVP, Upland, CA, USA).

### Statistics

Data are presented as the mean ± standard error of the mean. Statistical analysis was performed using one-way analysis of variance followed by Dunnett’s test for multiple comparisons. P<0.05 was considered to indicate a statistically significant difference.

## Results

### PBMC transplantation inhibited tumor growth

**Figure 1:**
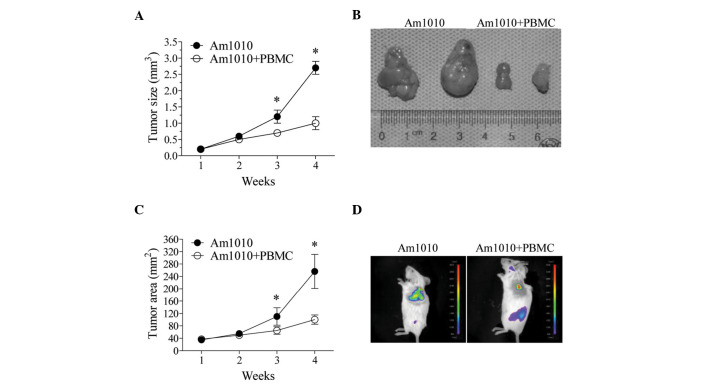
Tumor growth was observed after tumor segments were inoculated into the SCID/NOD mice, which were injected intraperitoneally human PBMCs or not. (A) The tumor growth curve by palpation, (B) tumors at the fourth week after inoculation, (C) the tumor area by fluorescence imaging and (D) tumor fluorescence imaging after inoculation. ^*^P<0.05, vs. the AM1010+PBMC group. SCID/NOD, non-obese diabetic/severe combined immunodeficient; PBMCs, peripheral blood mononuclear cells.

The inoculation of tumor segments produced 100% tumor growth in the NOD/SCID mice in the Am1010 and Am1010+PBMC groups. The tumor size increased with time (Fig. 1A), and when comparing tumor growth in the two groups, it was faster in the Am1010 group compared with the Am1010+PBMC group. The difference in tumor size between the two groups became significant from the third week after inoculation (P<0.05) (Fig. 1A,B).

We further examined the tumor growth with fluorescence imaging in the Am1010 and Am1010+PBMC groups. Under fluorescence imaging, the GFP protein of the tumors was expressed stably. The tumors appeared green-yellow at the inoculation sites (Fig. 1D). The fluorescence area in the two groups increased with time (Fig. 1C). It was significantly larger in the Am1010 group than in the Am1010+PBMC group at the third and fourth week (P<0.05, Fig. 1C,D). These results showed that PBMC transplantation exhibited a marked inhibitory effect on tumor growth.

### T-lymphocyte reconstitution in the peripheral blood

**Figure 2:**
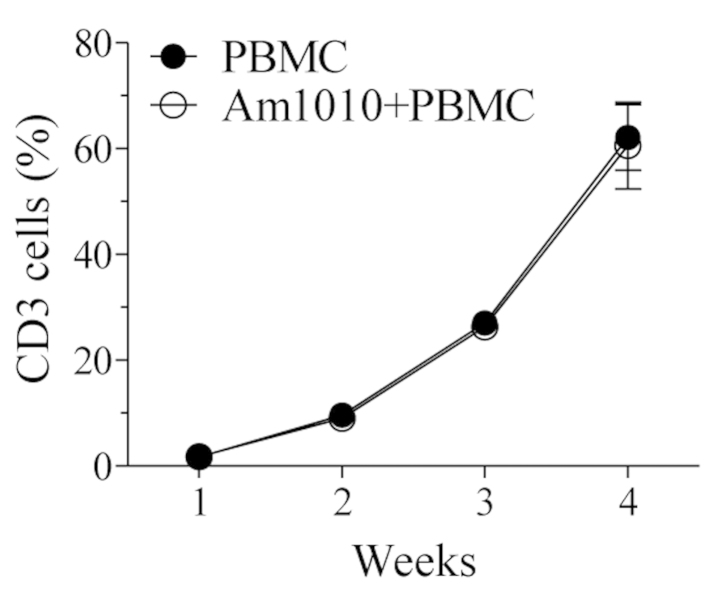
T lymphocytes in the peripheral blood. T-lymphocyte marker CD3 was measured with flow cytometry in PBMC-injected mice of the PBMC and AM1010+PBMC groups. The percentages of CD3^+^ cells among total nucleated cells are shown. SCID/NOD, non-obese diabetic/severe combined immunodeficient; PBMCs, peripheral blood mononuclear cells.

SCID/NOD mice are absent from T and B lymphocytes and NK cells. Previously, it has been shown that PBMC transplantation can reconstitute the immune system (15). To confirm this, we tested T-lymphocyte marker CD3 in the peripheral blood with flow cytometry. CD3^+^ cells were detected early at the first week after PBMC injection in the PBMC and Am1010+PBMC groups (Fig. 2). The ratio of CD3^+^ cells to normal human nucleated cells was more than 1% at the 1st week, and increased with time. The increase/trend was similar between the two groups. At the fourth week, the ratio of CD3^+^ cells was increased to 70%. Thus, the T-lymphocyte reconstitution was successfully carried out in this study.

### T-lymphocyte recruitment in the spleen

**Figure 3:**
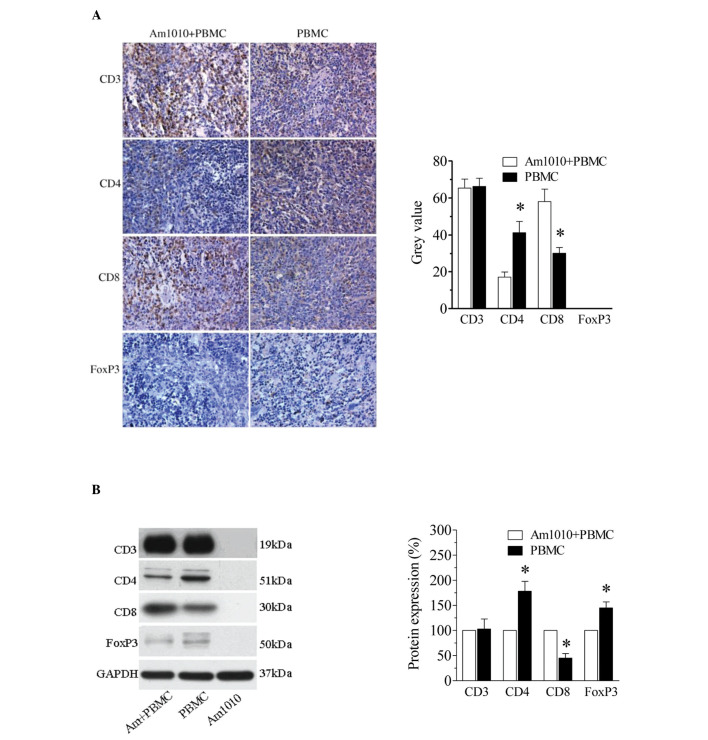
Recruitment of T lymphocytes in the spleen. The spleens were removed after four weeks of tumor segment inoculation and/or PBMC injection. The positive cells and expression levels of CD3, CD4, CD8 and FoxP3 were examined by (A) immunohistochemistry (magnification, ×200) and (B) western blotting. The gray value was shown. ^*^P<0.05, vs. the AM1010+PBMC group. PBMC, peripheral blood mononuclear cell.

To further examine the reconstitution of the immune system, we examined T-lymphocyte recruitment in the spleen at the fourth week following PBMC transplantation using immunohistochemistry (Fig. 3A). Numerous CD3^+^ cells were seen in the spleens in the Am1010+PBMC and PBMC groups. The number of CD3^+^ cells appeared similar between the two groups. The subtypes of T lymphocytes were further evaluated by examining CD4, CD8 and FoxP3 immunoreactivity in the spleens. Among these subtypes in the Am1010+PBMC group, there were more CD8^+^ cells and fewer CD4^+^ cells. By contrast, there were more CD4^+^ cells and fewer CD8^+^ cells in the PBMC group. FoxP3, a specific marker of regulatory T-lymphocytes, was not detected in either of the two groups.

These T-lymphocyte subtypes were further examined using western blotting (Fig. 3B). Consistent with the immunohistochemistry results, the expression of CD3 was similar between the Am1010+PBMC and PBMC groups. In the Am1010+PBMC group, the CD8 levels were higher (P<0.05), but the CD4 levels were lower (P<0.05), compared with those in the PBMC group. Notably, FoxP3 protein expression was observed in the two groups. The levels of FoxP3 were lower in the Am1010+PBMC group than in the PBMC group. No CD3, CD4, CD8 or FoxP3 protein expression was observed in the Am1010 group, which served as negative control to prove the successful reconstitution of the immune system.

### T-lymphocyte recruitment in tumors

**Figure 4:**
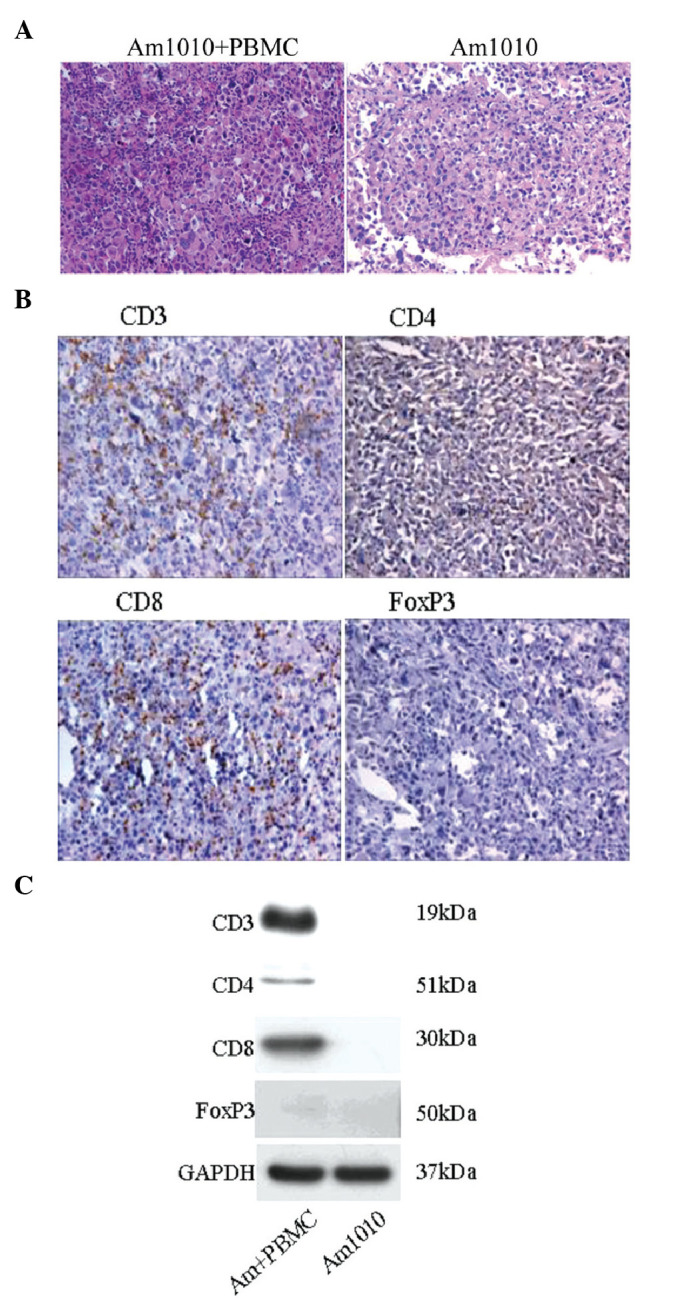
Recruitment of T lymphocytes in tumors. The tumors were removed after four weeks of tumor segment inoculation. (A) The tumor infiltrating cells were tested with hematoxylin and eosin staining (magnification, ×200). The positive cells and expression levels of CD3, CD4, CD8 and FoxP3 were examined by (B) immunohistochemistry (magnification, ×200) and (C) western blotting. PBMC, peripheral blood mononuclear cell.

We examined whether T lymphocytes were recruited in the tumor tissues. Firstly, the tumor tissues were examined using H&E staining (Fig. 4A). In the Am1010 group, the tumor cells had similar size and regular arrangement. In the Am1010+PBMC group, the tumor cells were of different sizes. Notably, there were numerous smaller cells surrounding them. The infiltrated smaller cells were analyzed using immunohistochemistry (Fig. 4B); a large quantity of CD3^+^ and CD8^+^ cells, and a small quantity of CD4^+^ cells were identified. No FoxP3^+^ cells were observed. The four T-lymphocyte markers in the tumor tissues were then evaluated using western blotting (Fig. 4C). The results showed that CD3, CD4 and CD8 were present in cells of the Am1010+PBMC group, but not in cells of the Am1010 group. No FoxP3 expression was identified in either of the two groups.

## Discussion

The poor prognosis of lung cancer is largely due to the fact that lung cancer cells are resistant to chemotherapy drugs and metastasis. The underlying mechanisms have been presumed to be closely associated with dysfunction of immune system, although they are yet not elucidated to date (21,22). In the present study, we established a heterotopic lung carcinoma model in NOD/SCID mice using multi-drug-resistant Am1010 cells, which were prepared from human lung adenocarcinoma. In the model, human PBMC transplantation produced a significant inhibitory effect on tumor growth, and was accompanied by numerous T lymphocytes, particularly CD8^+^ cells, in the peripheral blood, spleen and tumor tissue. Our results provide direct evidence for the important role of immune reconstitution from PBMCs in lung cancer regression.

The Am1010 lung carcinoma cell line is resistant to cisplatin, taxol, and gefitinib. The cancer cells grow and form tumors *in vivo*, when injected into nude mice (5). In the present study, Am1010 lung carcinoma cells were injected into subcutaneous tissues in NOD/SCID mice, successfully inducing heterotopic tumor growth. When fragments of these tumors were inoculated into recipient NOD/SCID mice, they grew rapidly. The results support our hypothesis that Am1010 lung carcinoma cells and tumor fragments are a useful tool for studying lung carcinoma.

The NOD/SCID mouse strain was developed by crossing SCID mice with NOD mice (12). It has been reported that the engraftment levels of human splenocytes and PBMCs in NOD/SCID mice are 5- to 10-fold higher than those in the classical SCID mice (23,24). Consistent with these reports, the present study showed that intraperitoneal injection of human PBMCs into the NOD/SCID mice produced a gradual increase of human CD3 lymphocytes in the peripheral blood, and the ratio of CD3 lymphocytes was ≤70% of the original amount at the fourth week. In addition, immunohistochemistry and western blotting revealed the presence of numerous CD3 cells in the spleen. Thus, the data demonstrate that PBMC transplantation successfully induces T-lymphocyte reconstitution in NOD/SCID, not only in the peripheral blood, but also in a lymphoid organ.

Numerous studies have analyzed the association between tumors and immunity. TILs have been widely reported to have protective functions against tumors. TILs have been identified in various types of tumors, including lung cancer. Their existence has been found to be associated with decreased tumor progression (25), induction of responses to chemotherapy (26,27) and increased lifetime of patients (28). TILs derived from neonatal cord blood mononuclear cells induced marked human lung and cervical tumor remission in NOD/SCID mice, and the antitumor effect was accompanied with a high infiltration of CD3^+^ T cells in tumors and a marked induction of apoptotic cell death (25). In the present study, PBMC engraftment by intraperitoneal injection significantly slowed the lung tumor growth and induced CD3^+^ T-lymphocyte recruitment in tumors. Therefore, the TILs may be an important factor for lung cancer remission in this study.

T lymphocytes are mainly composed of helper lymphocytes (CD4^+^ T cells), cytotoxic T lymphocytes (CTLs or CD8^+^ T cells) and regulatory T cells (FoxP3^+^ T cells) (29). A great quantity of animal studies and clinical data has revealed the important role of T-lymphocyte subtypes in antitumor effects (30,31). An investigation of 335 cases of non-small cell lung cancer (NSCLC) showed that an increased number of epithelial CD8^+^, stromal CD8^+^ and stromal CD4^+^ lymphocytes was significantly correlated with improved disease-specific survival. In particular, a low level of stromal CD8^+^ lymphocyte infiltration was associated with an increased incidence of angiolymphatic invasion (32).

CD8^+^ T cells have been found to be associated with improved outcome in the majority of human tumors (33), including lung cancer (32,34). In the present study, we observed that T lymphocytes infiltrated into tumors. The T-lymphocyte subtypes were mainly CD8^+^ lymphocytes. Thus, the CD8^+^ lymphocytes from PBMCs may underlie the key protective mechanism against lung cancer progression in this study.

In contrast to CD8^+^ lymphocytes, infiltration of tumors by regulatory T cells is instead associated with poor prognosis in NSCLC and other carcinomas (35). The regulatory T cells are thought to function primarily in cancers by repressing CD8^+^ T-cell function. The regulatory T-cell depletion may be therapeutically beneficial (35). In the present study, FoxP3, the marker of regulatory T cells, was not observed in tumors in immunohistochemistry and western blotting, although FoxP3 protein expression was observed in the spleen. The results suggest that PBMC-inoculated mice may have intact CD8^+^ T-cell function, which exerts potent tumor toxicity effect.

In conclusion, in the present study, we applied multi-drug-resistant lung carcinoma cells to explore the protective effect of immune reconstitution from PBMC transplantation. The infiltration of a high proportion of CD8^+^ T lymphocytes, but not regulatory T lymphocytes, into tumors may underlie the mechanism. To the best of our knowledge, this is the first study involving immunoprotection and multi-drug-resistant lung carcinoma in general. This study may provide a basis for immunotherapy in drug-resistant lung carcinoma.
